# Níveis Séricos do BDNF na Proteção Cardiovascular e em Resposta ao Exercício

**DOI:** 10.36660/abc.20201001

**Published:** 2021-03-03

**Authors:** Carla Paixão Miranda, Fernando Antônio Botoni, Manoel Otávio da Costa Rocha

**Affiliations:** 1 Universidade de Brasília BrasíliaDF Brasil Universidade de Brasília - Patologia Molecular, Brasília, DF - Brasil; 2 Universidade Federal Belo HorizonteMG Brasil Universidade Federal de Minas Gerais, Belo Horizonte, MG - Brasil

**Keywords:** Doenças Cardiovasculares/mortalidade, BDNF, Fator Neutrófico Derivado de Encéfalo, Endotélio Vascular, Fatores de Crescimento Neural, Polimorfismo, Exercício

Prezado editor,

Li com interesse o artigo publicado por Trombetta et al.^1^ Neste sentido, é cada vez mais sabido a importância da divulgação de novos marcadores na doença cardiovascular. Trago para discussão uma proteína chamada, fator de crescimento/diferencial 15 (GDF-15) que foi identificada pela primeira vez como citocina 1 ou MIC-1 da família do TGF-beta, apresenta efeitos pleiotrópicos dependendo do modelo a qual está sendo estudado.^2^ O interessante nessa discussão é a interface com que o Fator Neurotrófico Derivado do Cérebro (BDNF) interage com a molécula do GDF-15 que também apresenta efeitos no sistema vascular,^3^ porém em elevadas concentrações pode apresentar resposta adversa, considerado um marcador prognóstico independente para a doença cardiovascular há evidências que o exercício físico controla os níveis séricos de GDF-15 nos protegendo de doenças cardíacas/coronárias, doenças metabólicas e doenças oncológicas.^2^^,^^3^ Neste contexto, o BDNF que também é um marcador prognóstico na doença cardiovascular com função para além do receptor cerebral de maneira a promover a maturação do GDF-15.^4^ Segundo o racional teórico a proteína convertase subtilisina/cexina (PCSK) apresenta, em sua constituição, uma tríade com sequência de aminoácidos (Asp-His-Ser) com função catalítica promotora de clivagem do GDF-15 e maturação para o meio intracelular.^4^ Evidências mostraram que precursores de GDF-15 não clivados e em grandes quantidades na matriz extracelular se correlacionam com risco aumentado para um pior desfecho clínico na insuficiência cardíaca.^4^
