# Supplementary material for: Níveis Séricos do BDNF na Proteção Cardiovascular e em Resposta ao Exercício
Source: Arq Bras Cardiol. 2021 Mar 3;116(3):510. [Article in Portuguese] doi: 10.36660/abc.20201001 (PMC8159551; doi:10.36660/abc.20201001)
Supplement: Translation

# Translation: Serum Levels of BDNF in Cardiovascular Protection and in Response to Exercise

**Affiliations:** 1 BrasíliaDF Brazil Universidade de Brasília - Patologia Molecular, Brasília, DF - Brazil; 2 Belo HorizonteMG Brazil Universidade Federal de Minas Gerais, Belo Horizonte, MG - Brazil

**Keywords:** Cardiovascular Diseases/mortality, BDNF, Brain-Derived Neurotrophic Factor, Endothelium Vascular, Nerve Growth Factors, Polymorfism, Exercise

Dear Editor,

I read with interest the article published by Trombetta et al.^1^ In this sense, the importance of disseminating new markers in cardiovascular disease is increasingly known. I bring to the discussion a protein called growth factor / differential 15 (GDF-15) that was first identified as cytokine 1 or MIC-1 of the TGF-beta family, it has pleiotropic effects depending on the model being studied.^2^ The interesting thing about this discussion is the interface with which the Brain-derived neurotrophic factor (BDNF) interacts with the GDF-15 molecule, which also has effects on the vascular system, but in high concentrations it can present an adverse response, considered an independent prognostic marker for the cardiovascular disease there is evidence that physical exercise controls serum GDF-15 levels, protecting us from heart/coronary diseases, metabolic diseases, and oncological diseases.^2^^,^^3^ In this context, BDNF is also a prognostic marker in cardiovascular disease with function beyond the brain receptor in order to promote the maturation of GDF-15.^4^ According to the theoretical rationale, the proprotein convertase subtilisin/kexin 9 (PCSK-9) presents in its constitution a triad with an amino acid sequence (Asp-His-Ser) with a catalytic function that promotes cleavage of GDF-15 and maturation to the intracellular medium.^4^ Evidence shows that GDF-15 precursors that are not cleaved and in large amounts in the extracellular matrix correlate with an increased risk of a worse clinical outcome in heart failure.^4^
